# Defining Clinical Response Criteria and Early Response Criteria for Precision Oncology: Current State-of-the-Art and Future Perspectives

**DOI:** 10.3390/diagnostics7010010

**Published:** 2017-02-15

**Authors:** Vivek Subbiah, Hubert H. Chuang, Dhiraj Gambhire, Kalevi Kairemo

**Affiliations:** 1 Departments of Investigational Cancer Therapeutics, Division of Cancer Medicine, The University of Texas MD Anderson Cancer Center, Houston, TX 77030, USA; 2 Nuclear Medicine, The University of Texas MD Anderson Cancer Center, Houston, TX 77030, USA; HHChuang@mdanderson.org (H.H.C.); kalevi.kairemo@docrates.com (K.K.); 3 UT School of Public Health, Houston, TX 77030, USA; dhiraj12m81@gmail.com

**Keywords:** WHO criteria, RECIST, EASL, mRECIST, RECICL, irRC, PERCIST, MDA criteria, RANO criteria

## Abstract

In this era of precision oncology, there has been an exponential growth in the armamentarium of genomically targeted therapies and immunotherapies. Evaluating early responses to precision therapy is essential for “go” versus “no go” decisions for these molecularly targeted drugs and agents that arm the immune system. Many different response assessment criteria exist for use in solid tumors and lymphomas. We reviewed the literature using the Medline/PubMed database for keywords “response assessment” and various known response assessment criteria published up to 2016. In this article we review the commonly used response assessment criteria. We present a decision tree to facilitate selection of appropriate criteria. We also suggest methods for standardization of various response assessment criteria. The relevant response assessment criteria were further studied for rational of development, key features, proposed use and acceptance by various entities. We also discuss early response evaluation and provide specific case studies of early response to targeted therapy. With high-throughput, advanced computing programs and digital data-mining it is now possible to acquire vast amount of high quality imaging data opening up a new field of “omics in radiology”—radiomics that complements genomics for personalized medicine. Radiomics is rapidly evolving and is still in the research arena. This cutting-edge technology is poised to move soon to the mainstream clinical arena. Novel agents with new mechanisms of action require advanced molecular imaging as imaging biomarkers. There is an urgent need for development of standardized early response assessment criteria for evaluation of response to precision therapy.

## 1. Introduction

With the advent of precision medicine, there has been an exponential growth in the armamentarium of genomically targeted agents. Evaluating early responses to precision therapy is essential for ”go” vs. ”no go” decisions for these molecularly targeted drugs. Our understanding of tumor biology and approach to treat cancers has surged in the recent past and is evolving rapidly. This reflects on the growth in the development of molecularly targeted therapeutic approaches for cancer therapy. This change in development pattern of anti-cancer drugs is questioning traditional ways of assessing tumor response. There is growing evidence that a single response criteria may not be good enough for all type of solid tumors with different therapeutic classes. The standard response criteria needs a critical interrogation, cross-verification and update. Many new criteria are being developed to address these evolving paradigms. In this article we review the commonly used response assessment criteria. We present a decision tree to facilitate selection of appropriate criteria. We also suggest methods for standardization of various response assessment criteria. We also try to identify trends in the development of these criteria with regard to anatomical imaging, functional imaging, organ/system specific criteria and future directions in development of response assessment criteria’s.

## 2. Methods

We reviewed the literature using the Medline/PubMed database. We used the key words “response assessment”, “treatment evaluation”, “anti-cancer drugs response evaluation”, “treatment response”, “WHO* criteria”, “RECIST*”, “RECIST 1.1”, “EASL*”, “mRECIST*”, “RECICL*”, “irRC*”, “PERCIST*”, “EORTC* PET response criteria” “Choi criteria”, “MDA* criteria”, “Macdonalds criteria”, “RANO* criteria”, published up to 2016. Peer reviewed manuscripts, abstracts and articles judged most relevant to the goals of this report were reviewed to identify various response assessment criteria published so far. The relevant response assessment criteria were further studied for rational development, key features included: definitions of lesions, measurement rules, calculation rules, response determination rules, reporting guidelines, validation of the criteria, proposed use, current use, and acceptance by various entities. In addition, specific case illustrations of utility of early response imaging were undertaken. We reviewed the medical records of patients with advanced cancer who had functional imaging as part of their care. This study was performed in accordance with the guidelines of the MD Anderson Institutional Review Board (IRB). Because this was a retrospective chart review IRB has waived the consent requirements.

*Key:
WHO criteria: World Health organization
RECIST: Response Evaluation Criteria In Solid Tumors
EASL: European Association for the Study of the Liver
mRECIST: modified response evaluation criteria in solid tumors
RECICL: The Response Evaluation Criteria in Cancer of the Liver
irRC: immune-related response criteria
PERCIST: PET Response Criteria in Solid Tumors
EORTC PET response criteria: European Organization for Research and Treatment of Cancer
MDA criteria: MD Anderson criteria
RANO criteria: Response Assessment in Neuro-Oncology

## 3. Results

### 3.1. History: Development of Response Assessment Criteria

Initial effort to systematically define response assessment criteria on larger scale was made by WHO in 1979, which resulted in the WHO handbook for reporting results of cancer treatments [1]. Though the distinction of solid tumor like sarcoma was apparent at this time from hematological malignancies like leukemia, the distinction in response pattern within solid tumors was not obvious.

In the subsequent years, many groups like Southwest Oncology Group [2] tried to develop guidelines to fulfill demands for greater rigor in response and endpoint definitions. However, several problems in terms of interpretation of these criteria appeared, leading to numerous modifications or clarifications and resulting in a situation in which response criteria no longer were comparable among research organization [3].

This resulted in the need for an effort to review the existing guidelines. In 1994, several organizations involved in clinical research combined forces to tackle the review of these criteria on the basis of the experience and knowledge acquired since then. They proposed a new set of guidelines RECIST 1.0 [4]. These criteria enjoyed wide acceptance and greatly improved the standardization in recording and reporting the response objectively in solid tumors. Despite these advantages, it has been demonstrated that their applicability in different neoplasms is less than optimal. Several reports have been published regarding the low reliability of RECIST criteria in evaluating response in different types of tumors, such as prostate cancer, malignant pleural mesothelioma, non-small cell lung cancer, gastrointestinal stromal tumor, soft tissue sarcoma, neuroendocrine tumors, and disseminated pediatric malignancy [3].

With the development of new classes of drugs (cytostatic, anti-angiogenic, immunotherapy, etc.), wider availability of newer imaging modalities (PET scan, MRI, Nuclear imaging, etc.) and greater understanding of tumor biology; it became clear that one response assessment criteria may not be good fit for all solid tumors. RECIST criteria also do not take into account changes in various tumor characteristics apart from size like tumor viability, metabolic activity and tumor density that may be associated with tumor response.

This led the efforts by various specialized groups to define tumor specific response criteria. During first decade of 2000 various site specific and mechanism specific criteria were proposed (See Table 1). Meanwhile EORTC developed RECIST 1.1 to address updates on various measurement rules like number of lesions need to be assessed, use of RECIST in various clinical trial scenarios, utilization of newer imaging technologies such as 18-Fluoro-deoxyglucose positron emission tomography [FDG-PET] and MRI; assessment of lymph nodes; need of confirmation is truly needed; applicability of RECIST in trials of targeted non-cytotoxic drugs, etc.

However, the development of new criteria is necessitated and continued thereafter.

### 3.2. Response Assessment Criteria

Here in we review all the response criteria and discuss the strengths and weaknesses of the criteria. Table 1 provides an overview of all response criteria.

#### 3.2.1. WHO Criteria

WHO criteria was first large scale effort to standardize the response assessment and reporting in cancer clinical trials. It was published as “WHO handbook for reporting results of cancer treatments” (reference—handbook) and subsequently by Miller and colleagues in paper “Reporting Results of Cancer Treatment” [5]. The handbook and paper have reported requirement for essential data recording, criteria for response assessment and reporting guidelines. The principal driver for development of these criteria was to standardize the recording and reporting of cancer clinical trials.

WHO criteria provided detailed description of measurement of lesion. The lesions were classified into two groups as measurable and non-measurable depending upon ability to accurately and reproducibly measure the lesion. The size of the lesion was derived as two-dimensional measure with multiplication of longest diameter by its perpendicular diameter. Unidimensional measurements were allowed in certain conditions like hepatomegaly. Complete response, partial response, no change and progressive disease were defined separately for measurable and non-measurable disease and bone metastasis. The rules for determining overall response considering all lesions were described. The concepts of duration of response and disease free interval were described.

WHO criteria has been validated in many prospective randomized trials and enjoyed status of most widely used criteria for quite some time. However inadequate description of details of measurement rules and handling of exceptions lead to development of many modifications to WHO criteria in various trials leading to loss of comparability. Many of the newer criteria are developed as modification to WHO criteria. With development of RECIST criteria use of WHO criteria is widely replaced with RECIST.

#### 3.2.2. RECIST

In late 1994, may years after release of WHO criteria, several clinical research organizations came together to review new insights gained after WHO criteria. The participating group developed a model by which response rate could be derived from unidimentional measurement as compared to bidimentional measurement in WHO criteria. The new concept was presented as RECIST 1.0 guidelines [4]. The guidelines were subsequently revised and version 1.1 was released in 2008 [6]. These are probably most comprehensive set of guidelines detailing various aspects of data capture, processing, interpretation and reporting of response assessment in solid tumor. Table 2 provides summary of important features and major changes RECIST 1.0 to RECIST 1.1.

RECIST 1.1 has been validated on large data warehouses and in prospective clinical trials. It has been accepted by many investigators, cooperative groups, industry and government authorities in the assessment of treatment outcomes in solid tumor.

#### 3.2.3. Organ System Specific Response Criteria

##### MDA Criteria for Bone Metastasis

Bone metastases were initially considered non measurable lesions in early response assessment criteria like WHO and RECIST. This was because bone metastases are typically located in irregularly shaped bones and are difficult to measure. Monitoring tumor response in bone metastases is clinically important in the management of cancer. Hence, in 2004 Hamaoka et al. [14] at The University of Texas MD Anderson Cancer Center updated the International Union Against Cancer (UICC) and WHO bone response criteria by expanding radiographic assessment and incorporating both CT and MRI. They proposed new response assessment criteria (MDA criteria) for response assessment of bone metastasis. The MDA criteria can be used to assess therapeutic response in numerous types of bone metastases. The MDA criteria allows use of various radiologic techniques. Baseline images can be obtained by X-ray (XR), CT, MRI, or by some other modality. The imaging modality selected in follow up should be compared against the baseline images that most clearly define the bone metastases. Skeletal scintigraphy and SPECT Scans (SS) should be used only to support other imaging modalities for assessing tumor response. The recommend duration for follow up imaging is every 2–6 months.

The proposed response criteria include quantitative and qualitative assessments of the behavior of bone metastases. Assessment by PET or (SPECT) is not included in the definition of response assessment. Complete response is defined as complete fill-in or sclerosis of lytic lesion on XR and CT, disappearance of hot spots or tumor signal on SS, CT, or MRI, normalization of osteoblastic lesion on XR and CT. Partial response is defined as sclerotic rim about initially lytic lesion or sclerosis of previously undetected lesion on XR or CT, partial fill-in or sclerosis of lytic lesion on XR or CT or regression of measurable lesion on XR, CT, or MRI or regression of lesion on SS (excluding rapid regression), decrease in blastic lesion on XR or CT. Every lesion need not have regressed to qualify for partial response, but no lesion should have progressed. Stable disease is defined as no change in measurable lesion on XR, CT, or MRI, No change in blastic/lytic lesion on XR, CT, or MRI and No new lesion on XR, SS, CT, or MRI. Progressive disease is defined as increase in size of any existing measurable lesions on XR, CT, or MRI or New lesion on XR, SS (exclude flares), CT, or MRI or Increase in activity on SS (exclude flares) or blastic/lytic lesion on XR or CT. The original definitions of change in dimension from WHO criteria are retained, PR is defined as a decrease of ≥50% in the sum of the perpendicular measurements of any lesion and PD as an increase of ≥25% in this sum [18].

Subsequently, MDA criteria has been validated and compared with WHO criteria in a retrospective study with bone-only metastatic breast cancer [19]. The authors reported with the MDA criteria, there were significant differences in PFS between patients classified as responders and those classified as non-responders (*p* = 0.025), but with the WHO criteria, there were not [19]. Neither criteria distinguished responders from non-responders in terms of OS. MDA response criteria correlated better than WHO response criteria with clinical response assessment.

In a pilot prospective study both MDA and WHO criteria predicted PFS of patients with osseous metastases at 6 months but not at an earlier time point [20].

Vassiliou and Andreopoulos suggested MDA criteria may be improved by becoming more objective and accurate [21]. Starting with the application of CT for assessing bone metastases, it would be very useful if the bone density in regions of metastases is measured in Hounsfield units (HU) after delineation of affected bone areas [21].

##### Choi Criteria for Gastrointestinal Stromal Tumor (GIST)

Some of the initial work by Choi et al. [9] indicated that the RECIST 1.0 significantly underestimated the initial tumor response to imatinib in patients with metastatic GISTs. At the same time, it was noted that there are significant changes in tumor density, enhancing intratumoral tumor nodules, and tumor vessels on contrast enhanced CT images after imatinib treatment [9]. Meanwhile EORTC criteria was available for response assessment using PET scan for assessing tumor activity. Unfortunately, access to PET is still limited for patients with GISTs, and in some lesions, the glucose uptake before treatment is not sufficient to be detected by FDG-PET. The aim of developing this criteria was to develop criteria using CT scan as imaging modality and use various tumor characteristics beyond size measurement to quantitative response evaluation in GIST.

Choi criteria used a combination of the values of tumor size and tumor density on CT scan to define responses [9]. The response criteria were defined as CR = disappearance of all lesions, no new lesions. PR = a decrease in size (sum of longest diameter as defined by RECIST criteria) of ≥10% or a decrease in tumor density (HU) ≥ 15% on CT, no new lesions, no obvious progression of non-measurable disease. SD = response does not meet the criteria for CR, PR, or PD, no symptomatic deterioration attributed to tumor progression. PD = an increase in tumor size of ≥10% and does not meet criteria of PR by tumor density (HU) on CT, new lesions, new intra-tumoral nodules or increase in the size of the existing intra-tumoral nodules.

However, elaborate rules for definitions of lesions, measurement rules, calculation rules, response determination rules, reporting guidelines were not reported. This leaves room for variation in the implementation of the criteria.

Choi criteria has been validated using time to progression endpoint. The results also have been compared with RECIST, EORTC criteria by various groups with variable results. It is also being used in assessing response in metastatic renal cell carcinoma [22], high grade soft tissue sarcoma, solitary fibrous tumor [23] and hepatocellular carcinoma [24].

##### RANO Criteria for High-Grade Gliomas

In 1990, Macdonald et al. [15] published criteria for response assessment in high-grade gliomas. These criteria provided an objective radiologic assessment of tumor response and were based primarily on contrast-enhanced computed tomography (CT) and the two-dimensional WHO oncology response criteria using enhancing tumor area. These criteria also considered the use of corticosteroids and changes in the neurologic status of the patient.

However, it is increasingly apparent that there are significant limitations using only contrast-enhancing component of the tumor. For example, pseudoprogression, high response rate to anti-angiogenic agents, inability to capture recurrence with non-enhancing component of the lesion. To address these issues Wen et al. proposed new response criteria, commonly known as Revised Assessment in Neuro-Oncology (RANO) criteria [16].

RANO criteria has provided definitions and rules for standardization of imaging definitions, number of lesions, and definition of radiographic response. The sum of products of diameters (SPD) is calculated as products of maximal diameters and adding them together. The response s are defined in contrast enhancing lesions, non-enhancing lesions and new lesions. The responses are based on thresholds defined in WHO criteria. The overall response is defined using response in enhancing, non-enhancing, new lesions, use of corticosteroids and clinical status of the patient [16].

##### Response Assessment Criteria for Hepatocellular Carcinoma (HCC)—EASL, mRECIST, RECICL

Non-operative patients of HCC are considered for systemic and locoregional therapies (LRTs). LRTs have shown improved survival in patients with unresectable HCC by inducing necrosis and delaying progression of the disease [25]. This presents a unique problem in radiological assessment of response in HCC. Measurement of tumor load by simple bi-dimensional determinations of diameter is not accurate enough, since tumor necrosis due to treatment is not taken into account. Therefore, the estimation of the reduction in viable tumor volume is considered the optimal method to assess the local response to treatment [10].

To address this issue commonly used response assessment criteria like WHO and RECIST have been modified by various groups. The European Association for the Study of Liver (EASL) criteria is based on WHO criteria incorporating the concept of viable tumor tissue [10]. Similarly, the American Association for the Study of Liver Disease (AASLD) developed a set of guidelines modifying RECIST criteria and aimed to accommodate the concept of viable tumor tissue. These guidelines are named as modified RECIST (mRECIST) [11].

In 2009 Liver Cancer Study Group of Japan proposed revisions to Response Evaluation Criteria in Cancer of the Liver (RECICL) previously published in 1994 and 2004. This is most commonly used criteria in Japan [12]. The criteria considers the biological characteristics of HCC like tumor necrosis is regarded as a direct effect of treatment, tumors are measured in two dimensions, and the dense accumulation of lipiodol is regarded as necrosis. Additionally, in 2009, revision complete response with and without enough ablative margin is defined, timing at which the overall treatment effects are assessed is defined and 3 tumor markers including alpha-fetoprotein (AFP) and AFP-L3 and des-gamma-carboxy protein (DCP) were also added for the overall treatment response [12,13].

### 3.3. Functional Assessment Response Criteria

Quantitative ^18^F-FDG PET was introduced for the early sequential monitoring of tumor response of breast cancer in 1993. Since then, there has been growing interest in using ^18^F-FDG PET to quickly assess tumor response to therapy [7]. With increasing availability of PET imaging techniques and adequate evidence of usefulness of PET imaging in assessment of response to cancer therapy, new response criteria using PET CT were proposed. The EORTC PET response criteria were proposed in 1999 [17], subsequently PERCIST 1.0 was proposed in 2009 [7].

#### 3.3.1. PET Response Criteria in Solid Tumors (PERCIST)

In PERCIST, response to therapy is assessed as a continuous variable and expressed as percentage change in SUL peak (or sum of lesion SULs) between the pre- and post-treatment scans. A complete metabolic response is defined as visual disappearance of all metabolically active tumors. A partial response is considered more than a 30% and a 0.8-unit decline in SUL peak between the most intense lesion before treatment and the most intense lesion after treatment, although not necessarily the same lesion. More than a 30% and 0.8-unit increase in SUL peak or new lesions, if confirmed, is classified as progressive disease. A greater than 75% increase in total lesion glycolysis is proposed as another metric of progression [7]. The comparison between PERCIST and RECIST 1.1 is presented in Table 3 and the PERCIST response categories are presented in Table 4. 

#### 3.3.2. EORTC

In EORTC criteria standardization and rules were proposed on following headings; patient preparation, timing of [^18^F]-FDG PET scans, attenuation correction and dose of [^18^F]-FDG, methods to measure [^18^F]-FDG uptake, tumor sampling, reproducibility, definition of [^18^F]-FDG tumor response [26].

Progressive metabolic disease (PMD) to be classified as an increase in [^18^F]-FDG tumor SUV of greater than 25% within the tumor region defined on the baseline scan, visible increase in the extent of [^18^F]-FDG tumor uptake (20% in the longest dimension) or the appearance of new [^18^F]-FDG uptake in metastatic lesions. Stable metabolic disease (SMD) would be classified as an increase in tumor [^18^F]-FDG SUV of less than 25% or a decrease of less than 15% and no visible increase in extent of [^18^F]-FDG tumor uptake (20% in the longest dimension). Partial metabolic response (PMR) would be classified as a reduction of a minimum of 15% ± 25% in tumor [^18^F]-FDG SUV after one cycle of chemotherapy, and greater than 25% after more than one treatment cycle. Complete metabolic response (CMR) would be complete resolution of [^18^F]-FDG uptake within the tumor volume so that it was indistinguishable from surrounding normal tissue [17].

## 4. Mechanism of Action Dependent Response Assessment Criteria

### Immune Related Response Criteria (irRC)

A nontraditional approach of killing tumor cells is introduced as therapeutic option with the approval of immunotherapies for treatment of cancer by USFDA. This new approach also has unveiled new patterns of tumor response as compared to conventional cytotoxic therapies. During clinical trial program for ipilimumab, a fully human monoclonal antibody that blocks CTLA-4, four distinct response patterns were detected: immediate response, durable stable disease, response after tumor burden increase, and response in the presence of new lesions. The first two patterns are conventional, whereas the latter two are novel and specifically recognized with immunotherapeutic agents [8]. To accommodate these novel response patterns, new response assessment criteria—irRC were proposed.

The irRC allow for the assessment of tumor burden as a continuous variable, which considers index lesions identified at baseline together with new lesions as they may occur after treatment start. Only measurable lesions are taken into consideration. Measures are taken bidimensionally for each lesion. The sum of the perpendicular diameters (SPD) of index lesions at baseline is added to that of new lesions to calculate total tumor burden.

Response categories under irRC are defined as immune-related complete response (irCR), immune-related partial response (irPR), immune-related stable disease (irSD), and immune-related progressive disease (irPD) using the same thresholds to distinguish between categories as defined under standard WHO criteria.

Using irRC, the appearance of new lesions alone does not constitute irPD if they do not add to the tumor burden by at least 25%. Patients with new lesions but an overall tumor burden decrease qualifying for partial response (≥50% decrease) or qualifying for stable disease (<50% decrease to >25% increase) are considered to have irPR or irSD, respectively [27].

These new patterns are considered clinically meaningful because they appear to be associated with favorable survival [28]. Phase III studies using ipilimumab using irRC, have demonstrated improvement in overall survival of metastatic melanoma patients [29]. More recently, this criteria has been validated in PD1 inhibitor pembrolizumab as well [30].

## 5. Response Criteria: Standardization, Challenges and Pitfalls

**Figure 1:**
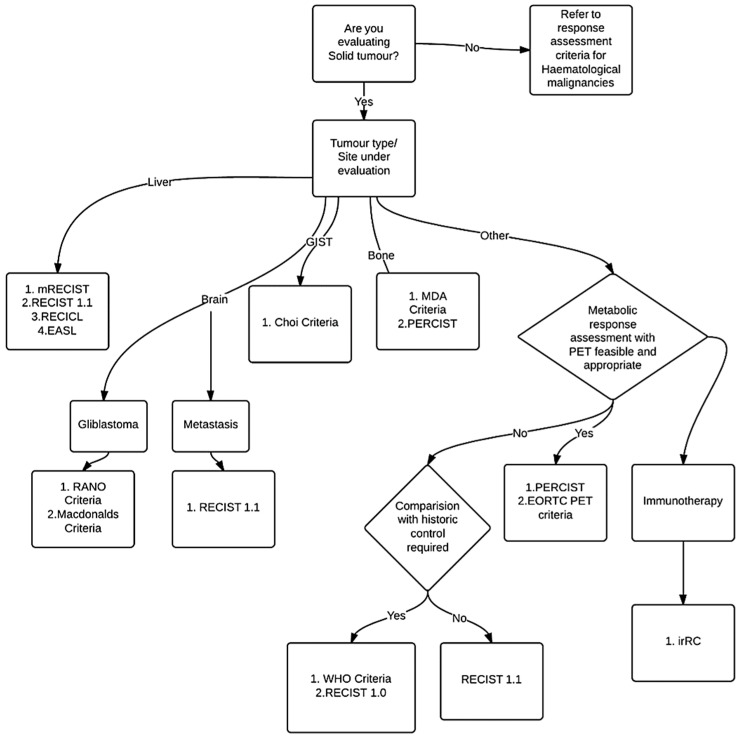
Decision algorithm for choosing response criteria.

General structure of response criteria: Some of the criteria like RECIST 1.1 are described very comprehensively including all steps required from data capture to reporting of the results. However, many others are not very comprehensive, leaving room for ambiguity and confusion. The general structure of the criteria can be defined and all groups can be requested to develop rules on those headings. The general structure can include clarification about Standardization of image acquisition, Definitions of lesions, Measurement rules, Calculation rules, Response determination rules, Reporting guidelines, Handling of exceptions, etc. Though designed with utmost precision, many of the criteria require subsequent clarification on various parameters. A formal mechanism to address confusion and periodic review shall be built in the process. In Figure 1 we present a decision algorithm for selection of the criteria.

Fixed part vs. Modifiable parts: Many of the aspects of the process are standard across different criteria and can be easily adapted for use in different criteria. Which parts of criteria can be standardized across all criteria to maintain a standard and determine which part can be customized shall be defined.

Methods of Validation: The requirement for validation of the criteria against existing standards and survival outcome should be defined. The criteria should be recommended for routine use only after appropriate validation steps.

## 6. Early Response Assessments and Imaging Biomarkers

Among the different modalities and the different response assessments discussed above, data exists in molecular imaging with FDG PET for early response assessments. There is some data in FLT-PET and NaF PET. A review [31] showed that FDG-PET is suitable in the early response assessment in breast cancer, lung cancer, colorectal cancer and lymphoma. At that time, the role of FDG in the early response assessment was questionable in head and neck cancer. However, recently, in a systematic review, the potential value of F-FDG PET/CT as a diagnostic tool for early response assessment in head and neck cancer patients has been confirmed [32].

Response to targeted therapy and ^90^Y embolization have also been demonstrated using FDG-PET in sarcomas [33,34] and Hodgkin’s lymphoma [35].

Timing of early response assessment and its definition is dependent on the type of therapy. There are not yet any guidelines or approved practice available, but some experimental data exists.

For example, the time point for [^18^F] FLT PET imaging of tumor response to gemcitabine is of crucial importance as lung carcinoma xenografts in mice after gemcitabine therapy. Early changes of [^18^F] FLT uptake in tumors reflected mechanisms such as competing gemcitabine uptake or gemcitabine-induced thymidylate synthase inhibition and only reflected growth inhibitory effects at a later time point [36]. Another study reported early response evaluation using NaF PET in osteosarcoma [37]. In Ewing sarcoma it has been shown that treatment response by quantitative [^18^F]-FDG PET assessed by PERCIST 1.0 as early as 9 days into insulinlike growth factor 1 receptor (IGF-1R) antibody therapy in patients with ESFT can predict the overall survival, progression free survival, and clinical response to therapy [38].

Recently, an imaging biomarker roadmap was presented for cancer studies which comprehensively reviews the field [39].

## 7. Case Studies of Early Response Assessments

Since FDG PET imaging has most data on early response evaluation we will illustrate some cases with this modality.

**Figure 2:**
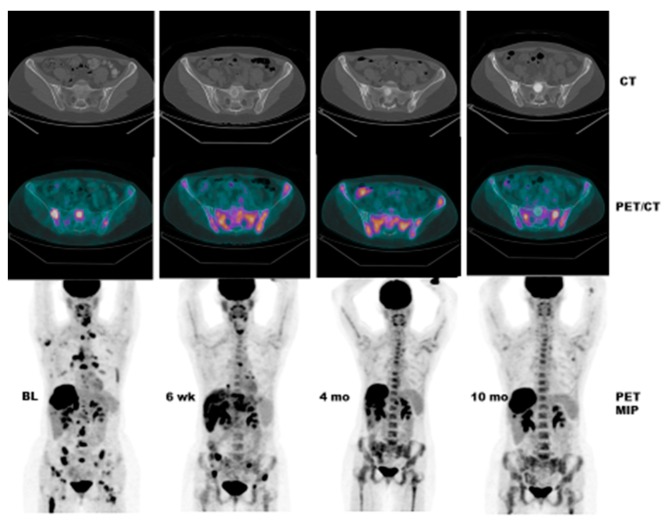
Pre- and Post FDG PET in a 47 y/o female with metastatic breast Cancer and multiple osseous metastases. Baseline study shows focal sites of activity in the bone marrow space, without discernible anatomic abnormality. About 6 weeks after starting therapy, there is diffuse marrow activation; however, there is relative loss of normal marrow activity where tumor was previously seen, and there is new sclerotic change at those sites. About 4 months after starting therapy, there is still diffuse marrow activation with loss of normal marrow activity where tumor was, and increased sclerosis on CT images. Unfortunately, about 10 months after starting therapy, she relapsed, with new focal sites of activity, without anatomic abnormality (similar to baseline study), whereas previously tumor sites have become densely sclerotic and remain without activity to suggest active tumor.

**Figure 3:**
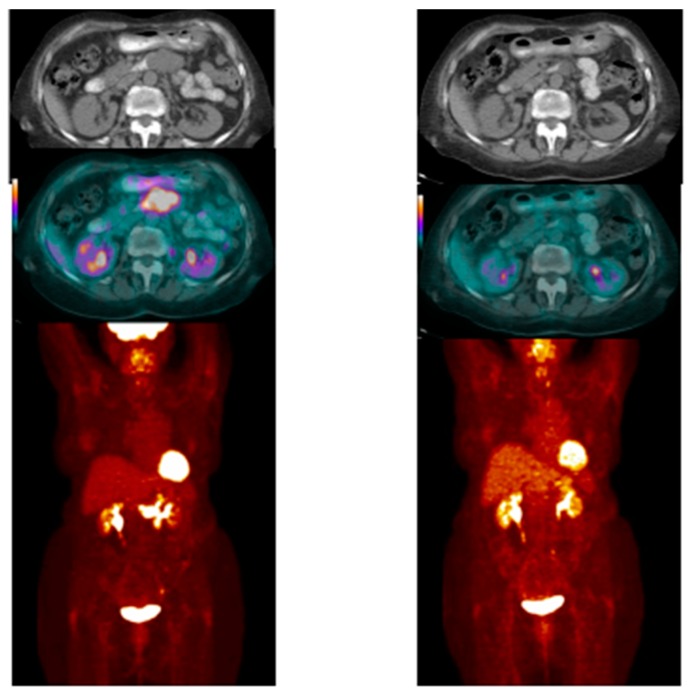
Pre- and Post FDG PET in an 82 old female with gastro-intestinal stromal tumor (GIST). Baseline FDG/PET study was performed off tyrosine kinase inhibitor therapy and she was initiated on a therapy. Repeat study was performed two weeks after initiation of Gleevec^®^ (Imatinib) a specific c-KIT inhibitor. Both anatomic and metabolic response was seen. Although there was still a residual anatomic abnormality, the tumor had complete metabolic response. This illustrates that the power of early functional imaging.

**Figure 4:**
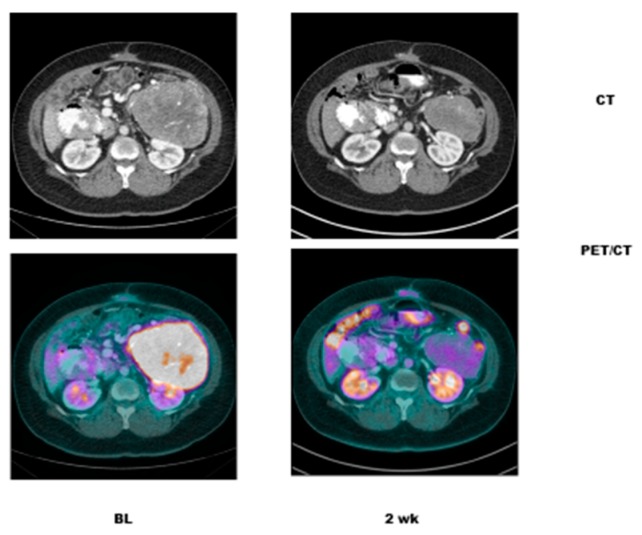
PRE and POST CT and PET/CT in a female in her 40’s with recurrent GIST. She has had multiple prior therapies and had relapsed from several TKI’s. After 2 weeks of novel therapy, she has had a complete metabolic response, but only a partial anatomic response.

**Figure 5:**
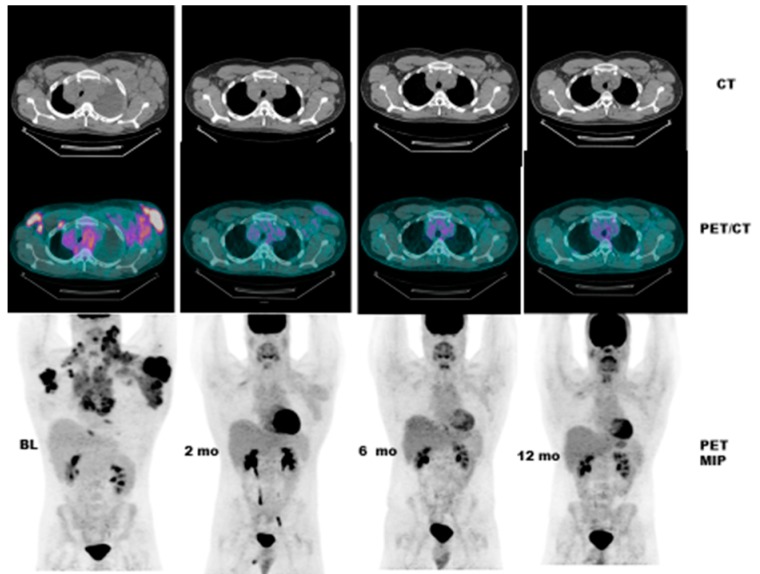
Pre and Post FDG PET CT in a 22 y/o male with Hodgkin lymphoma (nodular sclerosis type). He had complete metabolic response after 2 cycles of chemotherapy (ABVD) but only partial anatomic response. He completed 6 cycles of therapy with complete metabolic response but residual anatomic abnormalities. He had consolidation radiation therapy, about 1 month after completing chemotherapy; about 2 months after completing radiation therapy, there was no change. It is not uncommon to have residual masses after therapy for lymphoma, especially Hodgkin lymphoma, which may complicate anatomic response assessment.

Case # 1: FDG PET/CT was performed in a 47 y/o female with metastatic breast cancer. Patient had multiple osseous metastases. The baseline study showed focal sites of activity in the bone marrow space, without discernible anatomic abnormality (Figure 2). About 6 weeks after starting therapy, there was diffuse marrow activation; however, there was relative loss of normal marrow activity where tumor was previously seen, and there was new sclerotic change at those sites. About 4 months after starting therapy, there was still diffuse marrow activation with loss of normal marrow activity where tumor was, and increased sclerosis on CT images. Unfortunately, about 10 months after starting therapy, she relapsed, with new focal sites of activity, without anatomic abnormality (similar to baseline study), whereas previously tumor sites became densely sclerotic and remained without activity to suggest active tumor.
Case # 2: This is an 82 year old female with gastro-intestinal stromal tumor (GIST). Baseline FDG/PET study was performed off tyrosine kinase inhibitor therapy and she was initiated on a therapy. Repeat study was performed two weeks after initiation of Gleevec^®^ (Imatinib) a specific c-KIT inhibitor. Both anatomic and metabolic response was seen. Although there was still a residual anatomic abnormality, the tumor had complete metabolic response. This illustrates that the power of early functional imaging (Figure 3).
Case # 3: This is a 46 year old female with recurrent GIST. She had multiple prior therapies including imatinib, sunitinib, regorafenib, nilotinib, and pazopanib. After 2 weeks of new therapy with a novel TKI targeting c-KIT, she had a complete metabolic response, but only a partial anatomic response. This showcases early response can be seen as early as 2 weeks in GIST another sarcoma like Ewing’s sarcoma where in early responses as early as 9 days have been shown to predict survival [38] (Figure 4).
Case # 4: This is a 22 y/o male with Hodgkin lymphoma (nodular sclerosis type). He had complete metabolic response after 2 cycles of chemotherapy (ABVD) but only partial anatomic response. He completed 6 cycles of therapy with complete metabolic response but residual anatomic abnormalities. He had consolidation radiation therapy, about 1 month after completing chemotherapy; about 2 months after completing radiation therapy, there was no change. It is not uncommon to have residual masses after therapy for lymphoma, especially Hodgkin lymphoma, that may complicate anatomic response assessment [35,40,41,42] (Figure 5).

Implementation of early functional imaging assessments to targeted therapy could help in “go vs. no go” decisions in developmental therapeutics.

## 8. Response Assessments in Radiology

### Current State-of-the-Art

A standardized early response assessment criterion is crucial in precision oncology. In this review we have described several criteria which are used in clinical trials. During the evolution of response assessment criteria WHO and RECIST stand out as most important landmarks influencing overall development in this field. The primary intention of these criteria was to bring standardization of response assessment and reporting. It is quite a challenge that we still use different criteria and the heterogeneity within different groups. When we carefully look at the rational for development of various criteria to address specific situations, they appear perfectly justifiable. This situation leaves researchers puzzled about use of appropriate criteria in different situations. For investigators evaluating all the relevant evidence for appropriateness and accuracy of the criteria before use doesn’t seem realistic. Probably we need to change the paradigm of standardization from one standard criteria for all tumors to standardization of framework for response assessment criteria. Then based on the standard framework many different criteria can be developed by different expert groups which suites specific conditions. Once criteria are developed and validated using standard methodology its utility and acceptance will improve significantly and the need for customized criteria for different situations can be addressed.

## 9. The Future of “OMICS” in Radiology—Radiomics

Complementing the evolving landscape of “genomics” in targeted therapy a new field of “***OMICS***” in radiology—***RADIOMICS*** has opened up [43]. This involves high-throughput, advanced computing programs and digital data-mining. With this advanced technology it is now possible to acquire vast amount of high quality imaging data [43]. Radiomics is rapidly evolving and is still in the research arena. This has the potential to advance to areas beyond oncology. It is poised to move soon to the mainstream clinical arena and a potential to provide critical decision making in personalized medicine.

## 10. Conclusions

With the advent of precision medicine, there has been an exponential growth in the armamentarium of genomically targeted agents. Evaluating early responses to precision therapy is essential for “go” vs. “no go” decisions for these molecularly targeted drugs. Several criteria exist for evaluating response to different therapies. However, none of them are perfect and need amendments. There is an urgent need for development of standardized early response assessment criteria for evaluation of response to precision therapy.

## Figures and Tables

**Table 1:** Major response assessment criteria.

Number	Criteria	Development Rational	Brief Description	Reference
1	WHO Criteria	To develop a common language to describe cancer treatment and to agree on internationally acceptable general principles for evaluating data.	Recommendations have been developed for standardized approaches to the recording and reporting of cancer treatments. Objective definitions of response using reduction tumor volume were published using bidimensional approach.	WHO Handbook (1979), Miller, A.B. et al. (1981) [5]
2	RECIST	To review the response definitions in use and to create a revision of the WHO criteria that, far as possible, addressed areas of conflict and inconsistency.	Response rates were derived from unidimensional measurements of tumor lesions and sum of diameters.	Therasse, P. et al. (2000) [4]
3	RECIST 1.1	To address questions raised after extensive use of RECIST 1.0 and to newer developments in imaging technologies and targeted therapies.	This revision of the RECIST guidelines includes updates that touch on all points like fewer than 10 lesions can be assessed, how to apply RECIST in randomized phase III trials where progression, not response, is the primary endpoint, how to utilize newer imaging technologies such as FDG-PET and MRI; how to handle assessment of lymph nodes; whether response confirmation is truly needed; and, not least, the applicability of RECIST in trials of targeted non-cytotoxic drugs.	Eisenhauer, E.A. et al. (2009) [6]
4	PERCIST	To Propose quantitative PET response assessment guideline.	Qualitative and quantitative approaches to metabolic tumor response assessment with ^18^F-FDG PET and a draft framework for PET Response Criteria in Solid Tumors.	Wahl, R.L. et al. (2009) [7]
5	irRC	Novel criteria for the evaluation of antitumor responses with immunotherapeutic agents.	The core novelty of the irRC is the incorporation of measurable new lesions into “total tumor burden” and comparison of this variable to baseline measurements.	Wolchok, J.D. et al. (2009) [8]
6	CHOI Criteria	To determine if CT criteria could be used in quantitative response evaluation in GIST.	A combination of the values of tumor size and tumor density on CT (a 10% decrease in tumor size or a more than 15% decrease in tumor density at 2 months of treatment) were used.	Choi, H. et al. [9]
7	EASL	Recommendations for response evaluation in HCC by European Association for the Study of the Liver while using WHO criteria.	Measurement of tumor load by simple bi-dimensional determinations of diameter is not accurate enough, since tumor necrosis due to treatment is not taken into account. To address this concern method of estimation of the reduction in viable tumor volume was suggested.	Bruix, J. et al. (2001) [10]
8	mRECIST	Recommendations for response evaluation in HCC while using RECIST criteria.	To address limitations of anatomic tumor response metrics when applied to molecular-targeted therapies or locoregional therapies in HCC.	Lencioni, R. et al. (2010) [11]
9	RECICL	Response evaluation criteria solely devoted for HCC.	HCC specific criteria to address the direct effects of treatment on the hepatocellular carcinoma (HCC) by locoregional therapies such as radiofrequency ablation (RFA), transcatheter arterial chemoembolization (TACE) and molecular targeted therapies, which cause necrosis of the tumor in the clinical practice as well as in the clinical trials.	Kudo, M. et al. (2010) [12,13]
10	MDA Criteria	To develop a practical approach for diagnosis and assessment of bone metastasis.	The MDA criteria divide response into 4 standard categories (CR, PR, PD, and SD) and include quantitative and qualitative assessments of the behavior of bone metastases.	Hamaoka, T. et al. (2004) [14]
11	The Macdonald Criteria	New criteria based on modern scanning and a fuller appreciation of the influence of steroids on neurologic findings and brain tumor images.	These criteria provided an objective radiologic assessment of tumor response and were based primarily on contrast-enhanced computed tomography (CT) and the two-dimensional WHO oncology response criteria using enhancing tumor area (the product of the maximal cross-sectional enhancing diameters) as the primary tumor measure. These criteria also considered the use of corticosteroids and changes in the neurologic status of the patient.	Macdonald, D.R. et al. (1990) [15]
12	RANO criteria	To address significant limitations of McDonalds criteria, which only address the contrast-enhancing component of the tumor.	The criteria included new information provided by MRI like T1, T2 images, Standardization of imaging definitions and measurement rules.	Wen, P.Y. et al. (2010) [16]
13	EORTC PET response criteria	To summarize the status of the technique and recommendations on the measurement of [^18^F]-FDG uptake for tumor response.	The EORTC PET study group has proposed a common method of assessing tumor [^18^F]-FDG uptake and reporting of response data.	Young, H. et al. (1999) [17]

**Table 2:** Summary of major changes RECIST 1.0 to RECIST 1.1.

Parameter	RECIST 1.0	RECIST 1.1
Minimum size measurable lesions	CT: 10 mm spiral, 20 mm non-spiral Clinical: 20 mm Lymph node: not mentioned	CT: 10 mm Clinical: 10 mm (must be measurable with calipers) Lymph node by CT *: ≥^$^15 mm short axis for target ≥10^$^–<15 mm for non-target <10 mm is non-pathological
Overall tumor burden	Up to 10 target lesions, maximum 5 per organ	Up to 5 target lesions, maximum 2 per organ
Response criteria Lymph node	Not defined	For CR lymph nodes must be <10 mm short axis
Progressive disease	20% increase over smallest sum on study or new lesions	20% increase over smallest sum on study and at least 5 mm increase or new lesions
Response criteria non-target disease	“unequivocal progression” considered as PD	More detailed description of “unequivocal progression” it must be representative of overall disease status change, not a single lesion increase
Overall response	Table integrated target and non-target lesions	Additional table with non-target lesion only. Guidance on CR in face of residual tissue
Confirmation of response	For CR and PR criteria must be met again 4 weeks after initial documentation	Required only for non-randomized trials with primary endpoint of response
Reporting of response results	9 categories suggested for reporting phase II results	Divided into phase II and phase III. 9 categories collapsed into 5
Guidance for imaging	Limited	updated with detailed guidance on use of MRI, PET/CT

* Notes included on measurability of bone lesions, cystic lesions; ^$^ greater than and/or equal to.

**Table 3:** Comparison between RECIST 1.1 and PERCIST Response Evaluation Criteria in Solid Tumors (RECIST 1.1) *.

Response Category	Criteria
Complete response	Disappearance of all target lesions
	Reduction in short axis of target lymph nodes to <10 mm
Partial response	Decrease in target lesion diameter sum > 30% ^†^
Progressive disease	Increase in target lesion diameter sum > 20% ^‡^
	>5 mm increase in target lesion diameter sum
	New, malignant FDG uptake in the absence of other indications of progressive disease or an anatomically stable lesion, and confirmed on contemporaneous or follow-up CT
	Unequivocal progression of nontarget lesions
Stable disease	Does not meet other criteria ^‡^

* Measurements are based on the sum of the unidimensional measurement of the greatest diameter of a maximum 5 lesions; ^†^ Reference standard: baseline sum; ^‡^ Reference standard: smallest recorded sum. Table modified from [6].

**Table 4:** Positron Emission Tomography Response Criteria in Solid Tumors (PERCIST) *.

Response Category	Criteria
Complete metabolic response	Normalization of all lesions (target and nontarget) to SUL less than mean liver SUL and equal to normal surrounding tissue SUL
	Verification with follow-up study in 1 month if anatomic criteria indicate disease progression
Partial metabolic response	>30% decrease in SUL peak; minimum 0.8 unit decrease *
	Verification with follow-up study if anatomic criteria indicate disease progression
Progressive metabolic disease	>30% increase in SUL peak; minimum 0.8 unit increase in SUL peak *
	>75% increase in TLG of the 5 most active lesions
	Visible increase in extent of FDG uptake
	New lesions
	Verification with follow-up study if anatomic criteria indicate complete or partial response
Stable metabolic disease	Does not meet other criteria

* Primary outcome determination is measured on the single most active lesion on each scan (not necessarily the same lesion). Secondary outcome determination is the summed activity of up to 5 most intense lesions (no more than 2 lesions per organ). Abbreviations: SUL, standardized uptake value using lean body mass; TLG, total lesion glycolysis. Table modified from [7].
